# Higher breastfeeding performance index is associated with lower risk of illness in infants under six months in Ethiopia

**DOI:** 10.1186/s13006-015-0057-2

**Published:** 2015-11-27

**Authors:** Demewoz Haile, Sibhatu Biadgilign

**Affiliations:** Department of Reproductive Health, College of Medicine and Health Sciences, Bahir Dar University, Bahir Dar, Ethiopia; Independent Public Health Research Consultants, Addis Ababa, Ethiopia

**Keywords:** Breastfeeding, Index, Infants, DHS, Ethiopia

## Abstract

**Background:**

Breastfeeding performance index is an explanatory attempt to summarize key breastfeeding practices by summarizing the different dimensions of breastfeeding practices into a single summary variable. Breastfeeding performance index is used to assess optimal breastfeeding practices by constructing a single composite index that includes timely initiation of breastfeeding, prelacteal feeding, current breastfeeding status, bottle feeding, any liquid given(except medicine) in the last 24 h, formula given in the last 24 h, any solid food given in the last 24 h. This study aimed to assess optimal breastfeeding practices of 0–6 month infants using breastfeeding performance index (BPI) and its association with childhood illness in Ethiopia.

**Methods:**

A secondary data analysis was carried out based on the Ethiopia Demographic and Health Survey (EDHS) 2011 data. The BPI was created using seven components by giving equal weight for all components during scoring. The data were described using descriptive statistics and analyzed using multivariable logistic regression.

**Results:**

The prevalence of low, medium, and high BPI was 18.41, 57.96 and 23.63 % respectively. The mean BPI score was 4.38 (SD 1.25; 95 % CI 4.31, 4.45). Multivariable logistic regression analysis showed that those infants who had low BPI score were 2.22 times (AOR = 2.22; 95 % CI 1.20, 4.11) and medium BPI category had 2.15 times at higher odds (AOR = 2.15; 95 % CI 1.23, 3.75) of developing diarrhea compared to infants in the highest BPI category. Being in the lower BPI category was significantly associated with higher odds of having fever (AOR = 1.73; 95 % CI 1.06, 2.80). Being in the medium index category was also associated with higher odds of having short and rapid breaths (AOR = 2.02; 95 % CI 1.01, 4.04).

**Conclusion:**

More than 80 % of the infants did not receive optimal breastfeeding practices based on the Breastfeeding Performance Index. Lower BPI was statistically associated with diarrhea, fever and short and rapid breaths illness in the last 2 weeks. This study implicates the importance of optimal breastfeeding to reduce childhood illness.

## Background

Optimal breastfeeding practices rank among the most effective interventions to improve child health [1]. Studies showed that globally 60 % of infant and young child deaths attributed to lack of optimal breastfeeding practices, of which more than 13 % of under five deaths can be prevented by promoting exclusive breastfeeding [1, 2]. Lack of optimal breastfeeding practice is responsible for 45 % of neonatal infectious deaths, 30 % of diarrheal deaths and 18 % of acute respiratory deaths in children under 5 years [3]. In Ethiopia, about 18 % of the infant deaths have been attributed to poor feeding practices [4].

Annually, it is estimated that optimal breastfeeding of children under 2 years of age has the potential to prevent 1.4 million deaths in children under five of age in the developing world [5]. Optimal breastfeeding practices especially for infants < 6 months of age is not a single practice rather it constitutes several dimensions including timely initiation, colostrum feeding, exclusiveness, and whether or not the infant is given prelacteal feeds [6]. This multidimensional nature of optimal breastfeeding creates difficulty to assess its effect on health and nutrition of the infants. Measuring the effect of optimal breastfeeding for infants less than 6 months of age has been a great challenge because of absence of a single summary index that measures all the different breastfeeding recommendations of infants less than 6 months all at a time. For this reason in the ground, it is clearly need to develop composite indicators that can measure breastfeeding practices. Breastfeeding performance index (BPI) is an explanatory attempt to summarize key breastfeeding practices into single summary variable by summarizing the different dimensions of breastfeeding practices. According to study done by Senarath showed that creating a composite index to assess the overall breastfeeding performance among infants less than 6 months of age is feasible and applicable [7]. BPI can be effectively used to identify target groups for breastfeeding promotion interventions. The index could also be useful in establishing the association between breastfeeding and infant morbidity and evaluating the overall effect of interventions to promote breastfeeding. However, a detailed examination of the individual infant feeding indicators in the subpopulations with low BPI would still be needed when designing a public health interventions [7]. In addition, most previous researches on association between breastfeeding practices and health outcomes of infants have only considered the individual breastfeeding indicators like exclusive breastfeeding and bottle feeding practices.

Although there are studies about optimal breastfeeding practices in Ethiopia, there is no study which document optimal breastfeeding practices using composite summary index. This study aimed to assess optimal breastfeeding practices of infants less than 6 months of age using BPI and its association with childhood illness using 2011 Ethiopian Demographic Health Survey (EDHS) data.

## Methods

### Study setting and design

The sample for the 2011 EDHS was designed to provide population and health indicators at the national (urban and rural) and regional levels. The 2011 EDHS sample was selected using a stratified, two-stage cluster design and enumeration areas (EAs) were the sampling units for the first stage. A representative sample of 11,565 households was selected for the 2011 EDHS. However the analysis for this study was done for infants aged 0–6 months only. This is a quantitative cross-sectional study based on secondary data of Ethiopian DHS 2011. The detailed sampling methodology is presented in the DHS sampling of DHS country report [8].

### Data extraction

The Ethiopian DHS 2011 data were downloaded from Measure DHS website in SPSS format. After understanding the detail data, further coding of the data was done by the investigators. Data on a total of 1204 infants were included in the analysis and information on a wide-range of potential variables such as socio-demographic characteristics, economic variables, breastfeeding practices and childhood illness were extracted.

### Variable definition and measurement

Timely initiation of breastfeeding (≤1 h or > 1 h) was measured by asking the mother to recall when she had put her infants to her breast after delivery. Presence of prelacteal feeding (yes or no) was assessed by asking the mother to recall whether the indexed infant had taken anything other than breast milk within the third days of birth for some perceived benefit or cultural reasons. Bottle feeding practice (yes or no) was assessed by 24 h recall method whether the infant received something by bottle in the last 24 h. Current breastfeeding (yes or no) assessed by asking the mother to recall whether the infant breastfed in the last 24 h or not. Solid intake (yes or no), liquid intake (yes or no), formula intake (yes or no) was assessed by asking the mother to recall whether the infant received either of them in the last 24 h. Childhood illness i.e., diarrhea, cough, short and rapid breaths and fever (yes or no) of the infants was assessed by asking the mother to recall if the infant had any type of illness in the last 2 weeks preceding the survey.

### Breastfeeding performance index (BPI)

Breastfeeding performance index was constructed using seven items including timely initiation of breastfeeding, prelacteal feeding, current breastfeeding status, bottle feeding, any liquid given(except medicine) in the last 24 h, formula given in the last 24 h, any solid food given in the last 24 h. All inappropriate breastfeeding practices were scored as zero while recommended breastfeeding practices were scored as 1. The index was constructed using equal weight method as recommended by Senarath et al. for simplicity and understanding. Senarath et al. found that the index constructed using equal weight method and principal components analysis were highly correlated [7]. All the breastfeeding practices weigh equal value because this study considers all the feeding practices are equally important. There is currently neither standard guidance nor an empirical basis for weighting the various dimensions of infant and child feeding relative to one another. In the absence of such guidance, we gave equal weight to each component. The scores of each individual practice included in the index construction are then summed to give a possible range of 0–7 and the summed score divided in to three categories. The classification was based on a value recommendation of Arimond and Ruel on feeding index construction to minimize the differences between actual percentages and 33 %; when a choice is necessary, lump into the middle category so as not to dilute the contrast between the extremes [9]. The bottom 33 % of infants was referred to as ‘low BPI’, the next 34 % as ‘medium BPI’, and the top 33 % ‘high BPI. The BPI was divided into 3 categories (tertiles) in the following manner: a sum score of 0–3 categorized as low BPI, sum scores of 4–5 categorized as medium BPI, and sum scores of 6–7 were classified as high BPI. Those infants who fall in high BPI score were considered as optimally breastfed while infants with low and medium BPI score were considered as sub-optimally breastfed.

### Statistical analysis

Data were analyzed using STATA version 12. The “svy” command in STATA version 12 was used to weight the survey data. Descriptive statistics (mean, SD, percentages) were used to characterize the socio-demographic and childhood illness variables. Binary and multivariable logistic regression models were employed to determine the association between breastfeeding performance index and childhood illness. Variables which had statistically significant association in bivariate logistic regression at *p* value < 0.05 with childhood illness were considered to enter in the final multivariable model with BPI to control for their confounding effect on the association between BPI and childhood illness. Crude Odds Ratios (COR) and Adjusted Odds Ratios (AOR) were presented with 95 % confidence intervals. The association of BPI with childhood illness was declared significant at *p* value < 0.05 in the final multivariable logistic regression model.

### Ethical issues

The data were downloaded and used after the purpose of the analysis was communicated and permission was taken from Measure DHS Organization. The original DHS data were collected in confirmation with international and national ethical guidelines.

## Results

### Characteristics of the respondents

A total of 22.66 % of the households included in the analysis were found in the poorest wealth index category and 87.79 % of the household were residing in the rural area. About 45.78 and 11.32 % of the mothers had attended antenatal care (ANC) and institutional delivery for the indexed child respectively (Table 1).

**Table 1:** Characteristics of mothers having infants 0–6 months and their infants in Ethiopia: EDHS 2011

Variables	Weighted frequency	Weighted percentage
Wealth index		
Poorest	285	22.66
Poorer	279	22.17
Middle	287	22.75
Richer	220	17.49
Richest	188	14.93
Region		
Tigray	62	4.93
Affar	13	1.07
Amhara	256	20.31
Oromiya	583	46.22
Somali	45	3.57
Benishangul-Gumuz	14	1.10
SNNP	254	20.17
Gambella	4	0.32
Harari	2	0.18
Addis Ababa	23	1.81
Dire Dawa	4	0.32
Place of residence		
Urban	154	12.21
Rural	1107	87.79
Educational status of the mother		
No education	814	64.53
Primary education	380	30.20
Secondary education	54	4.28
Higher education	12	1.00
Sex of the infant		
Male	674	53.41
Female	587	46.59
Place of delivery		
Home	1,118	88.68
Health institution	142	11.32
Mode of delivery		
Vaginal delivery	1,239	98.25
Cesarean section	22	1.75
Antenatal visit		
Not attended	679	54.22
1–3 times	364	29.13
Four times and above	208	16.65
Maternal BMI		
<18.5	192	15.57
18.5–24.9	977	79.13
≥25	65	5.30
Maternal age category		
15–24	430	34.14
25–34	575	45.65
≥35	254	20.21
Birth order		
1	232	18.46
2–3	416	33.01
4–6	362	28.71
7^+^	250	19.82
Size of the child at birth		
Very large	208	16.54
Larger than average	143	11.40
Average	456	36.15
Smaller than average	126	10.02
Very small	325.	25.78
Had fever in last 2 weeks		
Yes	222	17.62
No	1,038	82.38
Had cough in last 2 weeks		
Yes	270	21.43
No	991	78.57
Problem in the chest or blocked or running nose^a^		
Chest only	45	26.34
Nose only	80	46.66
Both	44	25.56
don’t know	3	1.45
Short and rapid breaths^a^		
Yes	175	64.78
No	95	35.22
Diarrhea in the last 2 weeks		
Yes	132	10.44
No	1,129	89.56

^a^that is calculated for those who had cough only in the last 2 weeks

As shown on Table 1, about 17.62 % of infants had fever while 21.43 % the infants had cough in the last 2 weeks preceding the survey. Among those who had cough in the last 2 weeks preceding the survey, 26.34 % had chest problem, 46.66 % had nose problem and 25.56 % had both chest and nose problem. The prevalence of diarrheal diseases in the last 2 weeks preceding the survey was 10.44 %.

### Breastfeeding performance index

The breastfeeding performance index (BPI) scoring systems for infants 0–6 months include seven components (Table 2). The minimum BPI score was 0 while the maximum was 6. The mean score and standard deviation (SD) for the breastfeeding performance index was 4.38 (1.25; 95 % CI 4.31, 4.45). The BPI inter-quartile range scored from 4 to 5.

**Table 2:** Feeding practices among infants aged 0–6 months and breastfeeding performance index (BPI) scoring system, Ethiopia, 2011

Feeding practices	Weighted frequency	Weighted percentage	Score
Timely initiation of breastfeeding			
<1 h	564	44.93	1
>1 h	691	55.07	0
Pre lacteal feeding			
Yes	353	28.52	0
No	886	71.48	1
Current breastfeeding^a^			
Yes	1242	98.51	1
No	19	1.49	0
Bottle feeding^a^			
yes	196	15.57	0
No	1065	84.43	1
Liquids given^a^			
Yes	436	34.57	0
No	824.	65.43	1
Formula milk/ other milk^a^			
yes	212	16.82	0
No	1048	83.18	1
Solid foods given^a^			
Yes	123	9.74	0
No	1136	90.26	1

^a^24 hour recall. The total number is not equal due to missing values

**Fig. 1:**
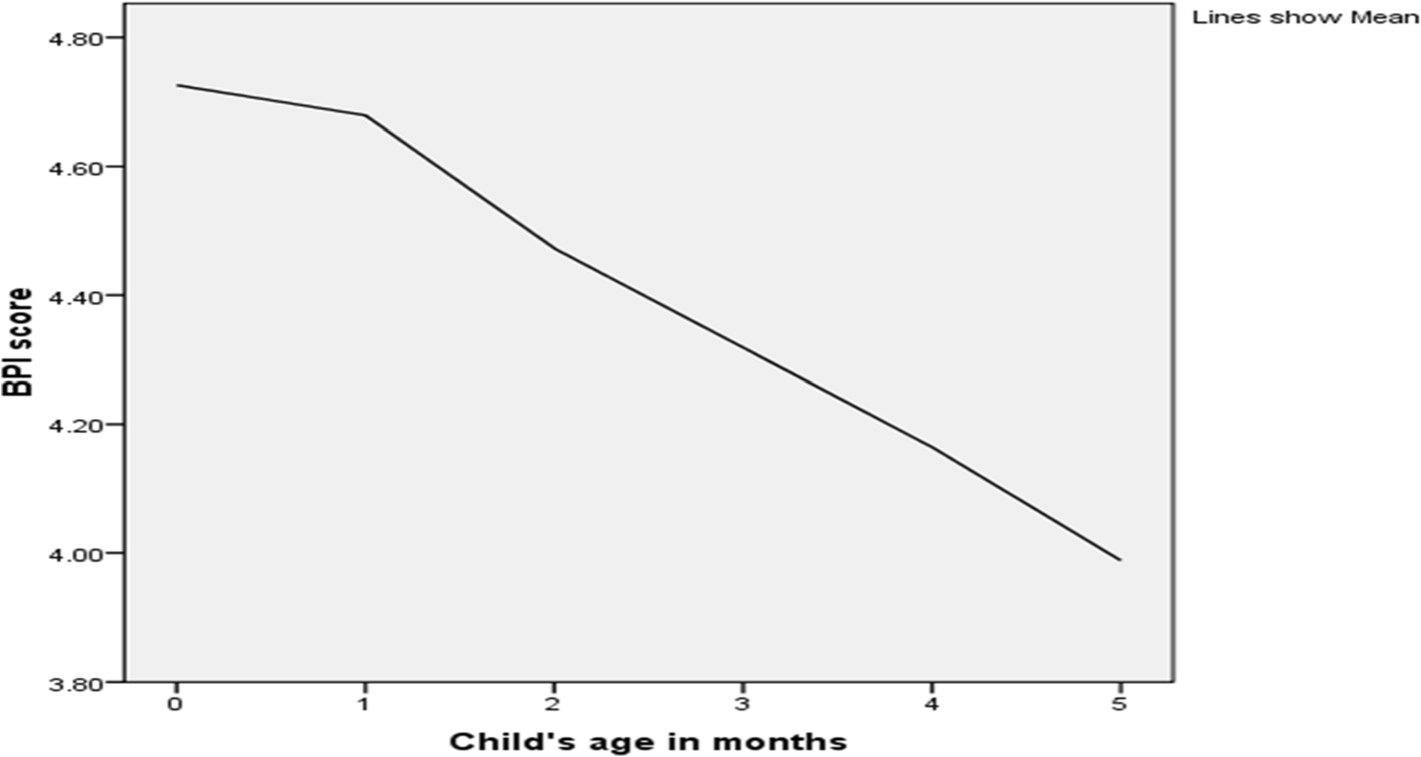
Breastfeeding performance index (BPI) score across the age infants in Ethiopia, 2011

Above half (44.93 %) of the respondents had no timely initiation of breastfeeding according to the Ethiopian infants and young child feeding (IYCF) guideline recommendation. Although 98.51 % of infants had breastfed in the last 24 h, 34.57 % of the respondents had another liquid given other than breast milk in last 24 h (Table 2). According to the BPI score, only 23.63 % of the infants had optimal breastfeeding practice (high BPI category) while the majority (57.96 %) of the infants were in the medium BMI index category. The breastfeeding performance index score decrease sharply when the age increases (Fig. 1).

In the binary logistic regression model, BPI was significantly associated with diarrhea, fever and short and rapid breaths illness in the last 2 weeks preceding the survey. However, cough symptom in the last 2 weeks was not significantly associated with BPI.

In the final multivariable model adjusted for the potential confounders, BPI was significantly associated with diarrhea, fever and short and rapid breaths symptoms in the last 2 weeks preceding the survey. Those infants found in the lowest and medium BPI category were 2.22 (AOR = 2.22; 95 % CI 1.20, 4.11) times and 2.15 (AOR = 2.15; 95 % CI 1.23, 3.75) times at higher odds to develop diarrhea as compared to those infants who found in the highest category, respectively. Those infants found in the lowest BPI category were 73 % at higher odds to have fever symptom in the last 2 weeks preceding the survey (AOR = 1.73; 95 % CI 1.06, 2.80). Those infants who found in the medium BPI category had 2.02 at higher odds to have symptoms of short and rapid breaths in the last 2 weeks preceding the survey (AOR = 2.02; 95 % CI 1.01, 4.04) (Table 3).

**Table 3:** Association between breastfeeding performance index and childhood illness status of 0–6 months infants in Ethiopia, 2011

BPI^b^	Childhood illness					
	Diarrhea		Short and rapid breaths	Cough in the last 2 weeks	Fever in the last weeks	
	COR (95 % CI)	AOR^a^ (95 % CI)	OR (95 % CI)	OR (95 % CI)	COR (95 % CI)	AOR^b^ (95 % CI)
Low	2.25 (1.22, 4.16)*	2.22 (1.20, 4.11)*	1.03 (0.49, 2.18)	1.44 (0.95, 2.20)	1.79 (1.11, 2.89)*	1.73 (1.06,2.80)*
Medium	2.18 (1.25, 3.79)*	2.15 (1.23, 3.75)*	2.02 (1.01, 4.04)*	1.01 (0.70, 1.47)	1.49 (0.97, 2.29)	1.44 (0.94, 2.21)
High	Reference	Reference	Reference	Reference	Reference	Reference

*Statistically significant at *p* < 0.05

^a^Adjusted for mothers’ and age

^b^adjusted for wealth index and birth order

## Discussion

The overall breastfeeding performance of mothers having infants less than 6 months of life was assessed by using breastfeeding performance index which comprises seven components. The prevalence of low, medium, and high BPI was 18.41, 57.96 and 23.63 % respectively. A study from Egypt reported that the proportions of infants belonging to low, average and high BPI categories were 27.1, 41.7 and 31.3 %, respectively [10]. The prevalence of low BPI category of this study is low as compared to the Egypt study. But in this study the prevalence of medium BPI category was high as compared to Egypt study. On the other hand, the prevalence of high BPI category was low as compared with the Egyptian study. This difference might be attributed to the difference in socio-cultural matter of childcare between the two countries.

According to this index the prevalence of optimal breastfeeding (high BPI) was 23.63 %. A recent study done in Ethiopia has documented that the prevalence of optimal breastfeeding as 24.6 % [11]. The mean score of the BPI was found 4.38 (±1.25) which is similar with the study done in Timor-Leste with mean BPI score of 4.4. In this study when we compare the prevalence of individual recommended components of BPI with the prevalence of high BPI, the prevalence of recommended individual practices of the index were higher. This could be because mothers do not practice all the recommended practices at the same level. This implies that using only one breastfeeding practices aspect cannot indicate the whole practice so that optimal breastfeeding could not be assessed by one dimension of breastfeeding only in infants less than 6 months of age. This study also showed that as age approaches to 6 months the overall BPI score deceases. This is because as age increases, it is expected that a large proportion of infants were no longer exclusively breastfed and/or receiving complementary feeding with bottle. This resulted significant lowering of the overall BPI score when the age increases.

Lower breastfeeding performance index was significantly associated with higher odds of having diarrhea. Those infants who found in the medium BPI category were 2.15 times at higher odds to have diarrhea illness as compared to those infants who found in the highest category. There were studies which showed that breastfeeding is associated with lower incidence of diarrhea [12–15]. A similar study conducted in Timor-Leste showed that high 2-week period prevalence of diarrhea was found in low BPI group as compared to the average and the high BPI group [7]. The odds across feeding groups showed a steadily increasing risk of diarrhea as the relative amount of breast milk decreased [16]. A pooled relative risk estimate from 23 studies showed that breastfeeding reduce the risk of diarrhea among infants ≤ 6 months [17]. This could be due to the immunological, hygienical and nutritional advantages of breastfeeding.

Breastfeeding performance index was also associated with lower fever symptom in the last 2 weeks preceding the survey. Being in the lower BPI category was 73 % at higher odds to have fever in the last 2 weeks. BPI was marginally associated with having a symptom of short and rapid breaths. Consistent finding has been also reported by other studies. Breastfeeding reduced the risk of hospitalization for respiratory infection by 57 % for infants ≤ 6 months [17]. Studies showed that breastfeeding was associated with lower incidence of respiratory tract infection [13, 14, 18] and lower BPI was associated with 2 week prevalence of acute respiratory infection in Timor-Leste [7].

### Public health implication

Breastfeeding has the single largest potential impact on child mortality of any preventive intervention like water and sanitation [19]. Breastfeeding is an effective child health intervention that does not require extensive health system infrastructure. Improvements in rates of exclusive and continued breastfeeding can contribute to the reduction of child mortality inequalities in developing countries [19]. The findings of this study support the hypothesis that good breastfeeding performance protects against infant morbidity. This implicates the importance of improving all dimension of breastfeeding in the first 6 months of life for preventing infants from illness. Along with this, it is clearly depicted that BPI may be useful to identify target groups for breastfeeding promotion interventions. The index could also be valuable in establishing the association between breastfeeding and infant morbidity and evaluating the overall effect of interventions to promote optimal breastfeeding.

The study has two basic limitations. The first limitation is that the study could not able to whether the morbidity results to score lower BPI or the morbidity comes because of the lower BPI. Recall bias and the secondary nature of data sources might impose some risk of bias for the actual estimation of the outcome of interest.

## Conclusion

More than 80 % of the infants did not experience optimal breastfeeding practice based on breastfeeding performance index. BPI was statistically associated with diarrhea, fever and short and rapid breaths illness in the last 2 weeks. This study implicates the importance of optimal breastfeeding to reduce childhood illness.
